# Investigation of Mechanical, Chemical, and Antibacterial Properties of Electrospun Cellulose-Based Scaffolds Containing Orange Essential Oil and Silver Nanoparticles

**DOI:** 10.3390/polym14010085

**Published:** 2021-12-27

**Authors:** Duy-Nam Phan, Muhammad Qamar Khan, Van-Chuc Nguyen, Hai Vu-Manh, Anh-Tuan Dao, Phan Thanh Thao, Ngoc-Mai Nguyen, Van-Tuan Le, Azeem Ullah, Muzamil Khatri, Ick-Soo Kim

**Affiliations:** 1 School of Textile-Leather and Fashion, Hanoi University of Science and Technology, 1 Dai Co Viet, Hanoi 10000, Vietnam; hai.vumanh@hust.edu.vn (H.V.-M.); tuan.daoanh@hust.edu.vn (A.-T.D.); thao.phanthanh@hust.edu.vn (P.T.T.); 2 Department of Textile and Clothing, Faculty of Textile Engineering and Technology, National Textile University, Karachi Campus, Karachi 74900, Pakistan; 3 School of Chemical Engineering, Hanoi University of Science and Technology, 1 Dai Co Viet, Hanoi 10000, Vietnam; chucnguyenvan85@gmail.com (V.-C.N.); mai.nguyenngoc@hust.edu.vn (N.-M.N.); 4 School of Mechanical Engineering, Hanoi University of Science and Technology, 1 Dai Co Viet, Hanoi 10000, Vietnam; tuan.levan@hust.edu.vn; 5 Nano Fusion Technology Research Group, Institute for Fiber Engineering (IFES), Interdisciplinary Cluster for Cutting Edge Research (ICCER), Shinshu University, Tokida 3-15-1, Ueda 386-8567, Nagano, Japan; 08tex101@gmail.com; 6 Department of Chemistry and Materials, Faculty of Textile Science and Technology, Shinshu University, Tokida 3-15-1, Ueda 386-8567, Nagano, Japan; muzamilkhatri@gmail.com

**Keywords:** cellulose nanofiber, silver nanoparticle, electrospinning, orange essential oil, antibacterial activity

## Abstract

This study demonstrated a controllable release properties and synergistic antibacterial actions between orange essential oil (OEO) and silver nanoparticles (AgNPs) incorporated onto cellulose (CL) nanofibers. The preparation of AgNPs attached on CL nanofibers was conducted through multiple processes including the deacetylation process to transform cellulose acetate (CA) nanofibers to CL nanofibers, the in situ synthesis of AgNPs, and the coating of as-prepared silver composite CL nanofibers using OEO solutions with two different concentrations. The success of immobilization of AgNPs onto the surface of CL nanofibers and the incorporation of OEO into the polymer matrix was confirmed by SEM-EDS, TEM, XRD, and FT-IR characterizations. The tensile strength, elongation at break, and Young’s modulus of the nanofibers after each step of treatment were recorded and compared to pristine CA nanofibers. The high antibacterial activities of AgNPs and OEO were assessed against Gram-positive *B. subtilis* and Gram-negative *E. coli* microorganisms. The combined effects of two antimicrobials, AgNPs and OEO, were distinctively recognized against *E. coli*.

## 1. Introduction

Cellulose is regarded as the most plentiful renewable bio-polymer on earth with the chemical structure of d-glucopyranosyl units joined by β-1,4-glycosidic bonds. Cellulose can be harvested through plants, wood, marine organisms, and biosynthesis for the manufacture of cellulose-based materials such as fibers, films, hydrogels, aerogels, and composites. Cellulose presents crystalline regions where the chains are tightly packed and with uniform cellulose molecular arrangements and amorphous domains with loose hydrogen bonding [1,2]. The rich hydroxyl groups on cellulose provide chemical modification to acquire cellulose derivatives and play a crucial role on controlling physicochemical properties of cellulose [3,4]. Cellulose nanofibers with excellent mechanical properties, highly thermostable due to the hydrogen bond intra macromolecules, have been used for biomedical applications, filtration, and food packaging [5,6].

Nanofibers are one-dimensional materials with promising applications such as in electronics, energy storage cells, catalysis, and filtering membrane [7,8]. The combination of polymers, metal, metal oxides, and other substances grants nanofibers multifunctional properties. Among many methods for nanofiber manufacture, electrospinning is a straightforward and versatile technique to fabricate nanofibers relying on electrostatic repulsion between charged polymeric solution and collector [9,10,11]. The method has been used to produce nanofibers or composite nanofibers with the diameters of tens to a few hundred nanometers. CL nanofibers fabricated using electrospinning method showcase Young’s modulus in the range of 120 to 170 GPa, 150 to 170 m^2^/g specific surface areas, high crystallinities, and thermal stability at temperatures up to 300 °C [12]. 

AgNPs have been extensively studied and used in the field of medicine in recent decades due to their broad spectrum of antibacterial, physical, chemical, and biological properties [13]. AgNPs have diameters in the range of few to 100 nm with a large surface-to-volume ratio, with low cytotoxicity for humans [14]. The mechanisms of silver antibacterial effects can be summarized as ion release, increased cellular oxidative stress, reactions with sulfur-containing cell membranes and phosphorus in DNA, and attraction of Ag to the negatively charged cell membrane of microorganisms [15,16]. Moreover, AgNPs are biocompatible with keratinocyte and fibroblasts and also display anti-inflammatory properties, even at very low concentrations [17].

Sweet orange is one of the most popular citrus fruits with fragrant taste and health promotion chemical compounds, largely available in many countries [18]. However, the peel is mostly discarded as biowaste or landfilling, which can result in environmental issues and is not economically desirable [19]. Orange essential oil has bioactive components that exhibit antioxidative, antimicrobial, and antiseptic activities against different pathogens and bacterial strains [20]. The use of essential oils (EO) in food and cosmetic industries is promoted due to health concerns of chemical substances [21]. 

Essential oil and silver-containing cellulosic scaffolds have valuable applications in drug delivery, release systems, medicine, and food packaging. Moreover, there are growing environmental concerns for synthetic polymers; therefore, biopolymers as cellulose and EO used as biodegradable scaffolds show great interest for the food industry and facial masks due to crack resistance, high porosity, and controllable compound release. In order to evaluate the feasibility of fabricating cellulose nanofibrous webs for applications related to antibacterial properties such as masks, wound dressings, food packaging, and protective clothing, we engineered AgNPs and OEO to the CL nanofiber scaffolds. The OEO contents were changed to explore the influence of OEO as coating material in CL composite nanofibers regarding chemical, mechanical, and antibacterial activities. 

## 2. Materials and Methods

### 2.1. Materials 

Cellulose acetate (average Mn = ≈50,000) was purchased from Sigma-Aldrich Chemical Co., Ltd. (St. Louis, MO, USA). Sodium hydroxide (NaOH, 97%), diethyl ether (99%), and silver nitrate (AgNO_3_, 99.8%) were acquired from Shanghai Aladdin Bio-Chem Technology Co., Ltd. (Shanghai, China). Acetone (99.5%) and N,N-dimethylformamide (DMF, 99.8%) were purchased from Wako Pure Chemical Industries, Ltd. (Osaka, Japan). Sodium borohydride (NaBH_4_, 95%) was obtained from BDH chemicals Ltd. (Poole, UK). Orange essential oil was extracted from the peel of Vietnam’s sweet orange (citrus sinensis) and purchased from local company. All chemicals were used as received without further purification. *Bacillus subtilis* ATTC 6633 and *Escherichia coli* ATCC 25922 were obtained from Microbiologics, Inc (Saint Cloud, MN, USA).

### 2.2. Silver and Orange Essential Oil Integrated in Cellulose Nanofibers 

**Scheme 1:**
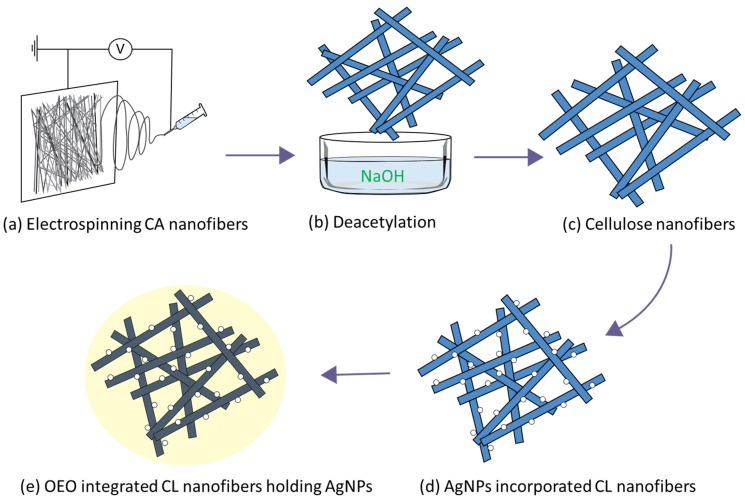
Schematic representation of the fabrication of orange essential oil-integrated cellulose nanofibers containing silver nanoparticles.

Cellulose nanofibers were prepared through two steps, electrospinning CA nanofibers and deacetylation process to convert CA nanofibers to CL nanofibers (Scheme 1). To prepare CA solution, we dissolved CA polymer in a bi-solvent system of acetone/DMF with the volume ratio of 1:1 and polymer weight percentage of 15 wt %. The solution was vigorously stirred for 12 h to dissolve the solute completely before being spun. The resultant CA solution was loaded into a 5 mL syringe with a metallic capillary needle of 0.6 mm internal diameter. The distance between the tip of the needle and the collector was fixed at 15 cm and the supplied voltage was 20 kV. The power supplier (HVU-30P100) has voltage and current capacity of 30 kV and 100 μA, respectively, and was purchased from MECC Co., Ltd., Fukuoka, Japan. The CA nanofibrous mats were collected after 24 h electrospinning. Afterward, the CA nanofibers were deacetylated by NaOH 0.01 M for 48 h. The acquired CL nanofibers were washed thoroughly with deionized water before being dried at 40 °C for 8 h, and eventually the immobilization process of AgNPs onto the CL nanofibrous webs occurred. 

To prepare AgNP-incorporated cellulose nanofibers, CL nanofibers were firstly immersed in 0.05 M AgNO_3_ solution for 1 h. The AgNO_3_-CL nanofibers were subsequently let to dry in an atmospheric environment for 24 h. The dried AgNO_3_-CL nanofibers were then dipped in NaBH_4_ 0.01 M solution for 1 h to let all chemical reactions occur completely; AgNO_3_ was reduced to AgNPs and entrapped into the CL nanofibrous webs. The composite Ag-CL nanofibers were collected, washed with DI water several times to remove all residual chemicals, and dried in air for 24 h. 

The OEO solutions were prepared with two concentrations of 5% and 10% (*v*/*v*) in ethanol. CL nanofibers were soaked in 10% OEO solution; the sample was denoted as OEO-CL nanofibers. Ag-CL nanofibers were soaked in 5% OEO and 10% OEO solutions for 1 h, and the collected samples were indicated as Ag-OEO5%-CL and Ag-OEO10%-CL nanofibers.

### 2.3. Characterizations

The qualitative chemical composition of OEO was identified using a gas chromatograph coupled with a single quadrupole mass spectrometry (Thermo Scientific model TRACE 1310 Gas Chromatograph with an ISQ 7000 Single Quadrupole Mass Spectrometer, GC-SQ-MS). The extract was prepared in diethyl ether. The experiment was performed by using a Perkin Elmer Clarus 500 gas chromatography equipped with an Elite-5ms capillary column (30 m × 0.25 mm × 0.25 μm) as well as mass detector turbo mass gold of the company, which was set in EI mode. The carrier’s gas flow was controlled at a rate of 1 mL/min. Simultaneously, the injector was managed at 250 °C. The oven temperature was programmed as follows: 50 °C at 5 °C/min (held for 5 min) to 140 °C at 7 °C/min and to 275 °C (held for 10 min).

The morphology and diameters of nanofibers were studied using scanning electron microscopy (SEM-JSM-6010LA, JOEL, Tokyo, Japan) at an accelerating voltage of 15 kV. The surface of the nanofibers was coated with platinum (Pt) in a sputtering chamber for 120 s at 30 mA. The fiber diameters with standard deviations were calculated using ImageJ application for 50 strands of fiber each sample. In addition, elemental analysis was studied on energy-dispersive X-ray spectroscopy (EDS).

The amorphous, crystalline, and semi-crystalline phases of all nanofibrous samples were investigated by X-ray diffraction instrument (XRD, Miniflex 300, Rigaku Co., Ltd., Tokyo, Japan) with Cu-Kα irradiation, operating at 30 kV and 500 mA, at the 2θ ranging from 5 ° to 80 °.

The characteristic vibration of functional groups present on the nanofibrous mats was analyzed using a Fourier transform infrared (FT-IR) spectrometer (Nicolet iS 50, FT-IR, Thermo, Waltham, MA, USA) in the range of 4000 to 400 cm^−1^ with accumulation over 20 scans.

The universal testing instrument (UTM, RTC-1250A, A&D Co., Ltd., Tokyo, Japan) was utilized to acquire stress–strain diagrams. The tensile test was performed five times for each sample, and the data were collected and used to calculate elongation at break, Young’s modulus, and tensile strength. Specimens were cut into a rectangular shape of 70 × 10 mm (length × width). The thickness of each specimen was measured before the pull testing, and the cross-sectional area was calculated by multiplying the thickness and the width. The working conditions of the instrument were 10 mm/min extensional rate and 50 mm gauge length.

Nanofibrous specimens were prepared in pieces with the size of 2 × 2 cm for the water absorption test. In detail, all specimens were weighed and then immersed in deionized water for 1 h. Afterward, the specimens were removed and gently placed on filter papers to discharge all dripping water. The specimens were weighed again, and water absorption amount was calculated by gained mass over the original mass of the specimens.

$$\mathrm{Water}\;\mathrm{absorption}\;(\%)=\frac{\left(W_{1}-W_{0}\right)}{W_{0}}\times 100$$

where *W*_1_ is the specimen weight after water immersion and *W*_0_ is the original weight of the specimen.

The silver release behaviors of silver-containing samples were examined in an aqueous environment using inductively coupled plasma mass spectroscopy (ICP-MS; Shimadzu ICPS-1000 IV; Shimadzu, Kyoto, Japan). In essence, 0.15 g of each silver-containing sample, Ag-CL, Ag-OEO5%-CL, and Ag-OEO10%-CL, were immersed in screw cap vials, each containing 50 mL deionized water, under gentle shaking and room temperature for 48 h. At various time intervals, 2 mL of liquid specimens were extracted and subjected to ICP-MS spectrometry to quantify silver concentrations. The release profiles were constructed by recording silver concentrations over a period of 48 h.

### 2.4. Antibacterial Assays

The antibacterial activities of CL as control sample, OEO-CL, Ag-CL, Ag-OEO5%-CL, and Ag-OEO10%-CL nanofibers were assessed against two bacterial strains, *E. coli* ATCC 25922 and *B. subtilis* ATTC 6633 in triplicate. The method was adopted from our previous publication [22]. In detail, *E. coli* was cultured in the LB liquid medium for 24 h at 37 °C, and *B. subtilis* was cultured likewise but at 30 °C. The cultured mediums were diluted by DI water to the concentration of 10^6^ colony forming unit per milliliter—CFU/mL. For the disk diffusion test, 200 µL of bacteria suspension was smeared over an agar plate. The nanofibrous specimens were cut into 5 mm diameter round shape and placed on agar plates seeded with bacterial cells in log phase. All plates were incubated at optimal conditions for bacterial growth (37 °C for *E. coli* and 30 °C for *B. subtilis*) for 24 h. All disk diffusion test processes were conducted in triplicate to measure the diameters of halo zones and calculate the standard deviations. 

## 3. Results and Discussion

### 3.1. Chemical Composition of the OEO

OEO was analyzed using GC-SQ-MS; the results showed the main constituents of OEO are d-limonene (64.33%), β-myrcene (8.84%), α-pinene (5.01%), α-phellandrene (2.82%), linalool (2.77%), decanal (1.89%), and octanal (1.28%) in 18.83, 17.29, 15.27, 16.72, 20.94, 24.2, and 17.68 (min), respectively (Table 1 and Figure S1). These seven compounds account for 86.94% of the total compounds identified. Limonene was also observed as the main component in the peel of sweet orange in several previous reports [23,24,25]. Among 36 volatile organic compounds detected, including monoterpenes, sesquiterpenes, aldehydes, alcohols, and esters, D-limonene was found to be the major contributor to the orange aroma. 

### 3.2. Morphology Study of Nanofiber Membranes

**Figure 1:**
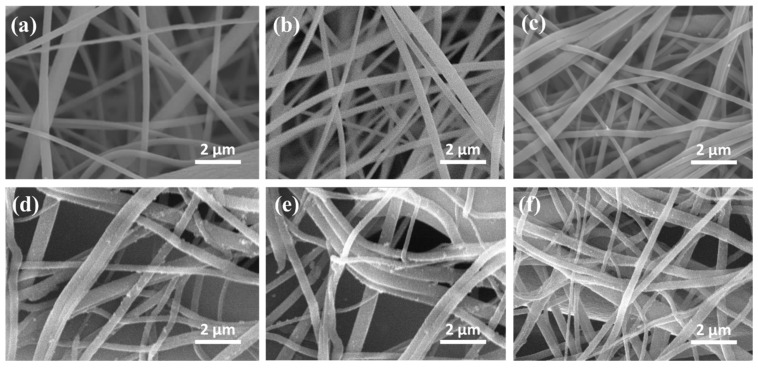
SEM images of (**a**) CA nanofibers, (**b**) CL nanofibers, (**c**) OEO-CL nanofibers, (**d**) Ag-CL nanofibers, (**e**) Ag-OEO5%-CL nanofibers, and (**f**) Ag-OEO10%-CL nanofibers.

In Figure 1a, CA nanofibers are observed to be diverse in size; the average diameter value with deviation was found to be 409 ± 157 nm. With the concentration of 15 wt % CA in bi-solvent system of acetone and DMF, the polymer chains entangled enough with each other to form a smooth and bead-free nanofibers. After treatment with NaOH, CL nanofibers presented similar morphology with slightly smaller diameter, 399 ± 134 nm, due to the mercerization effects, which induced the nanofibers to be contracted in diameter according to previous studies [26,27]. Similarly, OEO-CL nanofibers did not present any significant difference compared to CL nanofibers, and the presence of OEO could not detected due to the evaporation of essential oil in the vacuum chamber of SEM equipment. In Figure 1d–f, the AgNPs could also be spotted as being attached to CL nanofibers. The average diameters of these nanofibers increased to 430 ± 109, 426 ± 128, and 432 ± 85 nm due to the treatment process of AgNP immobilization and the incorporation of OEO; AgNPs physically attached to the nanofibrous network structure. The morphology of Ag-OEO5%-CL and Ag-OEO10%-CL presented similar morphology compared to the Ag-CL sample because OEO was completely removed in the vacuum environment of the microscope chamber. 

### 3.3. FT-IR Spectral Analysis 

**Figure 2:**
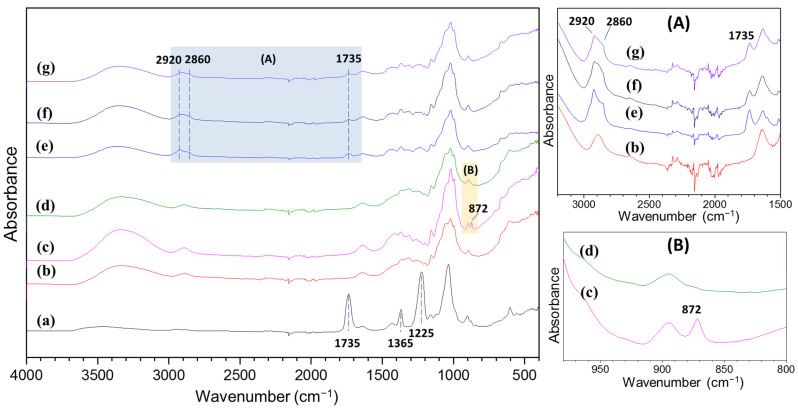
The FT-IR spectra of (**a**) CA, (**b**) CL, (**c**) AgNO_3_-CL, (**d**) Ag-CL, (**e**) OEO-CL, (**f**) Ag-OEO5%-CL, and (**g**) Ag-OEO10%-CL; (**A**) magnified view of FTIR spectrum of OEO; and (**B**) magnified view of FTIR spectrum of AgNO_3_.

Figure 2 presents the FT-IR spectra of pristine CA and CL nanofibers, and composite CL nanofibers after various steps of treatment. For CA spectrum, the strong absorbance peaks at 1735 cm^−1^, 1365 cm^−1^, and 1225 cm^−1^ corresponded to C = O vibration, C-CH_3_ vibration, and C-O-C stretching vibration, respectively [28,29,30]. After the deacetylation reaction, the complete disappearance of the peaks at 1735 cm^−1^ and 1225 cm^−1^ and the raising band at 3400 cm^−1^ proved the successful conversion of CA to CL. The addition of OEO gave rise to the peaks at 2920 cm^−1^ and 2860 cm^−1^ assigned to -CH stretch, and 1735 cm^−1^ was attributed to carbonyl stretch of alkanes (Figure 2a) [31]. The peak at 872 cm^−1^ of AgNO_3_-CL spectrum was assigned to -NO_3_ bending, which can be referred to the penetration of silver nitrate into the cellulosic polymer matrix. This peak was removed after the redox reaction between AgNO_3_ and NaBH_4_ (Figure 2b), attesting to the conversion from silver salt to AgNPs. 

### 3.4. X-ray Diffraction Study

**Figure 3:**
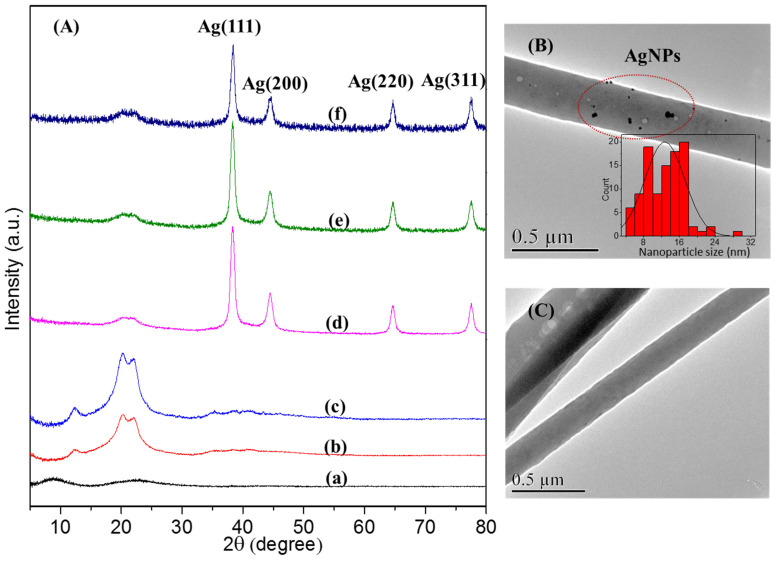
(**A**) XRD spectra of (**a**) CA; (**b**) CL; (**c**) OEO-CL; (**d**) Ag-CL; (**e**) Ag-OEO5%-CL; and (**f**) Ag-OEO10%-CL. (**B**) TEM image of Ag-CL nanofiber. (**C**) TEM image of CL nanofibers.

The XRD patterns of CA and CL nanofibers, and AgNPs were identified using XRD technique. CA nanofibers exhibited two typical diffraction bands centered at 2θ of 9° and 22.5°, which corresponded to the semi-crystallinity of cellulose acetate polymer (Figure 3A(a)). The deacetylation process transformed CA to CL nanofibers completely, which showed typical bands of cellulose II. With the addition of OEO into the CL nanofibers, no characteristic bands or peaks could be detected. The presence of AgNPs attached to CL nanofibers can be confirmed by diffraction peaks at 2θ values of 38°, 44.5°, 64.5°, and 77.5° indexed to (111), (200), (220), and (311) crystal planes of the standard metal silver pattern, respectively [32,33]. The reduction of silver ions to AgNP formation from the nucleation thus were successfully conducted. As shown in Figure 3B, the TEM image of Ag-CL illustrates the adhesion of AgNPs on to the surface of CL nanofibers with the AgNPs’ diameter of 12.8 ± 4.4 nm. In contrast, neat CL nanofibers presented a particle-free morphology. The coating of Ag-CL nanofibers with OEO did not affect the X-ray diffraction (Figure 3A(e),(f)). 

### 3.5. EDS Analyses

**Figure 4:**
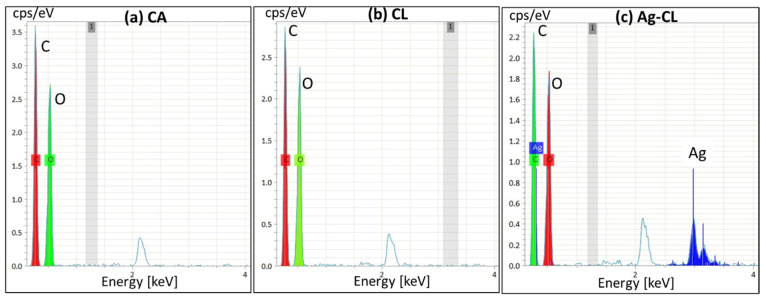
EDS spectra of (**a**) CA, (**b**) CL, and (**c**) Ag-CL nanofibers.

The elemental analysis was carried out by EDS study (Figure 4). The characteristic results verified that CA and CL nanofibers are composed majorly of carbon and oxygen. For the Ag-CL nanofibers, besides the presence of carbon and oxygen, the detection of silver demonstrated the loading of silver in the samples. Moreover, the nitrogen was not detected in Ag-CL, which verified the complete conversion of AgNO_3_ to AgNPs during the reducing reaction. The elemental composition is given in Table 2, CA and CL nanofibers were found to be composed of carbon, oxygen, and hydrogen elements. In the case of Ag-CL, the silver element was 3.64%, clarifying the success of AgNPs synthesis. 

### 3.6. Mechanical Properties

**Figure 5:**
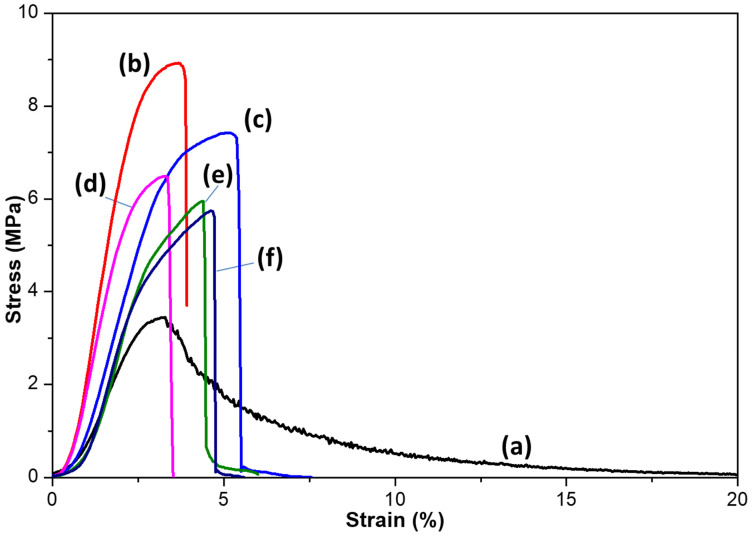
Stress–strain profiles of (**a**) CA, (**b**) CL, (**c**) OEO-CL, (**d**) Ag-CL, (**e**) Ag-OEO5%-CL, and (**f**) Ag-OEO10%-CL.

Table 3 and Figure 5 show the mechanical properties of CA, CL, OEO-CL, Ag-CL, Ag-OEO5%-CL, and Ag-OEO10%-CL. The deacetylation process and mercerization effect increased the strength at break and Young’s modulus of as-spun nanofibers from 3.32 ± 0.84 MPa and 144.31 ± 37.74 MPa to 8.51 ± 1.93 MPa and 315.51 ± 45.81 MPa, which are the values of CL nanofibers, respectively. The effect was due to the -OH groups of CL nanofibers, which form hydrogen bonding between inner polymer chains and possibly bind different nanofibers together. The bonding formation led to the void space reduction and tightly packed effect, increasing crystallinity and making the nanofibrous mats stronger and tougher [34]. The incorporation of OEO to CL nanofibers decreases the tensile strength but increases the elongation at break of nanofibrous samples, possibly the integration of essential oil loosens the intermolecular interactions between the polymer chains. Moreover, it could also be explained as the coating effect of OEO freed the linking nodes between different nanofiber strands and helped them move more easily in the tensile tests. However, after treating CL nanofibers with AgNO_3_ and NaBH_4_, the Ag-CL nanofibers present poorer mechanical strength and elongation at break. The adverse observation demonstrated the negative effect of AgNPs formation on the interconnective fiber network and the mobility [35,36].

### 3.7. Water Absorption and Silver Release Profile

**Figure 6:**
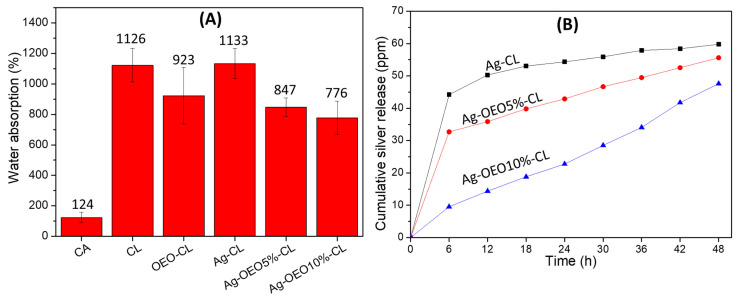
(**A**) Water absorption of CA, CL, OEO-CL, Ag-CL, Ag-OEO5%-CL, and Ag-OEO10%-CL. (**B**) Silver release behavior of Ag-CL, Ag-OEO5%-CL, and Ag-OEO10%-CL.

Water absorption was evaluated to quantify the water amount can be absorbed into CA nanofibers, as well as CL nanofibers before and after the incorporation of silver and OEO (Figure 6A). The water holding capacity of CL nanofibers after deacetylation process improved significantly and reached to 1126% compared to CA nanofibers at just 124%. For composite nanofibers, the quantification of water absorption gives insight into the influence of AgNPs and OEO to the hydrophilicity of treated CL nanofibers. The percentage of weight gain of CL nanofibers and Ag-CL nanofibers showed a slight difference, which means the adhesion of AgNPs did not significantly change the hydrophilic properties of the CL nanofibers; AgNPs were considered hydrophobic but the added percentage of silver content proved to be insufficient to change the absorption properties of CL nanofibers. By contrast, OEO addition reduced the water absorption of the CL nanofibers from 1126% to 923% for OEO-CL, and from 1133% to 847% and 776% of Ag-CL, Ag-OEO5%-CL, and Ag-OEO10%-CL, respectively. These findings were in line with other publications [37,38]. 

The mechanisms of AgNP toxicity are closely related to Ag^+^ ion release from the polymer matrix into the cell, and controlling the release of silver over time is desirable in wound dressing and other medical applications. Long-term silver release can lead to long-lasting antibacterial activity. The silver release has been reported to correlate to silver content, AgNPs size, water chemistry, and the surface coating of AgNPs. In Figure 6B, AgNP dissolution was studied over a time course of 48 h. Ag-CL presented a speedy release at first, followed by a gradual release curve. The silver discharge of Ag-OEO5%-CL was slower in the first 6 h compared to Ag-CL, and followed by a stable increase, whereas the discharge profile of Ag-OEO10%-CL showed a first-order linear relationship. It is surmised that the phenomenon was ascribable to surface passivation that occurred in the case of Ag-OEO5%-CL and Ag-OEO10%-CL due to the OEO coating effects. The rapid discharge of Ag at the 6 h time point of Ag-CL was in accordance with other already published articles, which could be explained as the discharge of chemisorbed Ag^+^ and the oxidative process with O_2_ [39]. The coating effects of OEO cause the slow and sustained release of Ag from the composite nanofibers [40,41].

### 3.8. Antibacterial Activity of Composite CL Nanofibers

**Figure 7:**
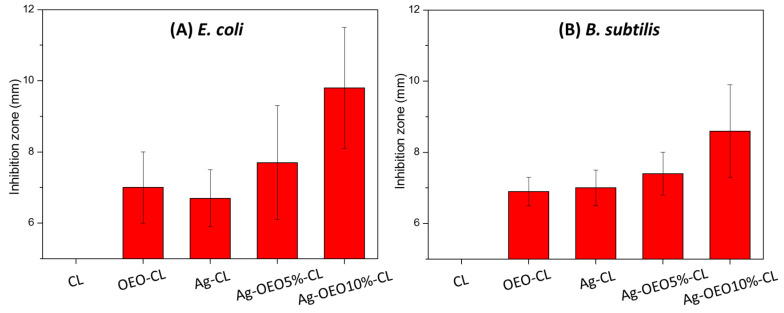
Diameter of inhibition zone with standard deviation of CL, OEO-CL, Ag-CL, Ag-OEO5%-CL, and Ag-OEO10%-CL against (**A**) *E. coli* and (**B**) *B. subtilis*.

The antibacterial effects of CL, OEO-CL, Ag-CL, Ag-OEO5%-CL, and Ag-OEO10%-CL were evaluated against common microorganisms, *E. coli* and *B. subtilis*. The photos of the inhibition zones were taken after incubation time and measured using ImageJ software. Figure 7 and Figure S2 show the diameters of the halo area, generated by the disruption of bacterial growth around the nanofibrous specimens. The CL nanofiber sample or negative control sample presented no antibacterial activities. For all other samples containing AgNPs or OEO, the inhibitions zones were clear and visible, demonstrating the antimicrobial properties against both Gram-positive and Gram-negative bacteria. In the case of *E. coli*, the zone diameters were 7.0 ± 1.0, 6.7 ± 0.8, 7.7 ± 1.6, and 9.8 ± 1.7 mm for OEO-CL, Ag-CL, Ag-OEO5%-CL, and Ag-OEO10%-CL. The combination effects of OEO and AgNPs resulted in improved antibacterial action against *E. coli*, and the sizes of the halo zones were significantly extended from 6.7 ± 0.8 to 9.8 ± 1.7 mm for Ag-CL and Ag-OEO10%-CL, respectively. However, OEO at low content was not so potent against *B. subtilis*—Gram-positive bacteria, when combined with AgNPs, adding OEO to Ag-CL composite nanofibers, the diameters of the halo zones increased from only 7.0 ± 0.5 to 7.4 ± 0.6 for Ag-CL and Ag-OEO5%-CL, respectively. The consistent results were also reported in a previous publication because of the bactericidal effects of OEO on diversities of bacterial cell structures, and OEO is less potent against Gram-positive bacterial strains [42]. OEO coating suppresses the silver release, thus leading to higher OEO concentrations, but the low silver discharge will not effectively extend the sizes of the bacterial inhibition zones against Gram-positive bacteria. The antibacterial properties of AgNPs can be assigned to the reactive oxygen species generation, destroying cell membrane and disturbing the DNA replication and protein synthesis. On the other hand, one of the most important mechanisms of essential oil antibiotics is disturbing the lipid and protein interactions and sabotaging their functions on the basis of their hydrophobicity. The highest inhibition zone was by CL-OEO10% against *E. coli*, which has complex structure of cell wall composed of a thin peptidoglycan layer. In a mixture, EO can interact with other compounds or antibiotic agents, producing antagonistic, additive, indifferent, or synergistic effects [43,44]. 

## 4. Conclusions

In recent years, customer interest in environmentally friendly, safe, and natural products has risen. Natural antimicrobial essential oils have antiviral, antimicrobial, and fungicidal benefits; high levels of safety; and a very low impact on human health, with great potential to replace synthetic chemical agents. The drawback can be remedied by combining them with fibrous structures and other metallic antibiotics such as silver, copper, or zinc oxide, which have been reported to offer many favors such as chemical and physical stability. There is some research on essential oils integrated into nanofibers; however, there are still many issues that need to be addressed such as the impacts of oil on mechanical, chemical, and overall performance. Moreover, the synergism when combined with silver is unclear and not yet well reported.

In the present paper, the success of AgNP in situ synthesis and the coating of OEO onto the CL nanofibers was introduced. The good antibacterial properties of AgNPs combined with OEO were demonstrated against *E. coli* and *B. subtilis*. The use of OEO as the coating material represents a sustainable and controllable silver release over the period of 48 h. Interestingly, the antibacterial synergism was great in the case of *E. coli*, whereas OEO content difference did not affect the bactericidal effectiveness against *B. subtilis*. It is concluded that OEO and AgNPs incorporated into CL nanofibers may have the potential as a biomaterial for avoiding bacterial infections in applications such as facial masks, protective clothing, and food packaging. However, more research is needed to further apply EO and AgNPs in commercialized products.

## Figures and Tables

**Table 1:** GC-SQ-MS-analyzed chemical compounds of OEO.

No.	Retention Time (min)	Compounds	Composition (%)
1	15.27	α-Pinene	**5.01**
2	16.72	α-Phellandrene	**2.82**
3	17.29	β-Myrcene	**8.84**
4	17.68	Octanal	**1.28**
5	18.83	D-Limonene	**64.33**
6	20.69	2-Carene	0.57
7	20.94	Linalool	**2.77**
8	21.45	p-Cymene	0.13
9	21.7	2-Caren-4-ol	0.34
10	22.14	cis-p-Mentha-2,8-dien-1-ol	0.93
11	22.27	(+)-(E)-Limonene oxide	0.42
12	22.61	Citronellal	0.37
13	23.26	1-Indanone, 4,5,6,7-tetrahydro	0.28
14	23.92	α-Terpineol	0.71
15	24.2	Decanal	**1.89**
16	24.73	2-Cyclohexen-1-ol, 2-methyl-5	0.63
17	25.08	Carveol	0.32
18	25.32	Neral	0.32
19	25.52	(−)-Carvone	0.74
20	26.14	Citral	0.76
21	26.45	2-Caren-10-al	0.31
22	28.15	Limonene oxide, trans-	0.16
23	29.37	Copaene	0.41
24	29.71	γ-Muurolene	0.46
25	29.88	Dodecanal	0.39
26	30.63	Caryophyllene	0.31
27	30.83	Copaene	0.43
28	32.47	Caryophyllene	0.82
29	32.71	Butylated hydroxytoluene	0.86
30	33.1	Cadina-1(10),4-diene	0.42
31	33.72	α-Acorenol	0.18
32	37.04	α-Longipinene	0.39
33	38.24	α-Sinensal	0.22
34	39.76	Nootkatone	0.15
35	42.35	m-Camphorene	0.16
36	43.05	1-Heptatriacotanol	0.1
Total			99.23

**Table 2:** Elemental composition of CA, CL, and Ag-CL nanofibers using EDS analysis.

Sample	Element (at.%)			
	Carbon	Nitrogen	Oxygen	Ag
CA	54.67	-	41.90	-
CL	52.44	-	47.56	-
Ag-CL	44.09	-	52.28	3.64

**Table 3:** Tensile strength, elongation at break, and Young’s modulus of CA, CL, OEO-CL, Ag-CL, Ag-OEO5%-CL, and Ag-OEO10%-CL nanofibers.

Sample	Tensile Strength (MPa)	Elongation at Break (%)	Young’s Modulus (MPa)
CA	3.32 ± 0.84	17.33 ± 5.79	144.31 ± 37.74
CL	8.51 ± 1.93	3.85 ± 0.98	315.51 ± 45.81
OEO-CL	7.83 ± 3.08	5.23 ± 2.38	254.95 ± 50.16
Ag-CL	6.32 ± 2.81	3.63 ± 1.25	220.27 ± 66.08
Ag-OEO5%-CL	5.91 ± 0.93	4.5 ± 1.91	215.94 ± 30.15
Ag-OEO10%-CL	5.6 ± 1.03	4.96 ± 1.65	204.9 ± 40.48

## Supplementary Materials

The following are available online at https://www.mdpi.com/article/10.3390/polym14010085/s1, Figure S1. The total ion chromatogram of OEO. Figure S2. Representative photographs of inhibition zone of CL—as the negative control, OEO-CL, Ag-CL, Ag-OEO5%-CL, and Ag-OEO10%-CL.
