# Supplementary material for: Investigation of Mechanical, Chemical, and Antibacterial Properties of Electrospun Cellulose-Based Scaffolds Containing Orange Essential Oil and Silver Nanoparticles
Source: Polymers (Basel). 2021 Dec 27;14(1):85. doi: 10.3390/polym14010085 (PMC8747631; doi:10.3390/polym14010085)
Supplement: Supplementary file 1 [file polymers-14-00085-s001.zip › polymers-1488923-supplementary.pdf]

polymers

MDPI

Supporting Information

Investigation of Mechanical, Chemical, and Antibacterial Properties of Electrospun Cellulose-Based Scaffolds Containing Orange Essential Oil and Silver Nanoparticles

Duy-Nam Phan [1,*], Muhammad Qamar Khan [2,*], Van-Chuc Nguyen [3], Hai Vu-Manh [1], Anh-Tuan Dao [1], Phan Thanh Thao [1], Ngoc-Mai Nguyen [3], Van-Tuan Le [4], Azeem Ullah [5], Muzamil Khatri [6] and Ick-Soo Kim [5,*]

[1] School of Textile-Leather and Fashion, Hanoi University of Science and Technology, 1 Dai Co Viet, Hanoi 10000 Vietnam; hai.vumanh@hust.edu.vn (H.V.-M.); tuan.daoanh@hust.edu.vn (A.-T.D.); thao.phanthanh@hust.edu.vn (P.T.T.)

[2] Department of Textile and Clothing, Faculty of Textile Engineering and Technology, National Textile University, Karachi Campus, Karachi 74900, Pakistan

[3] School of Chemical Engineering, Hanoi University of Science and Technology, 1 Dai Co Viet, Hanoi 10000, Vietnam; chucnguyenvan85@gmail.com (C.N.V.); mai.nguyenngoc@hust.edu.vn (N.-M.N.)

[4] School of Mechanical Engineering, Hanoi University of Science and Technology, 1 Dai Co Viet, Hanoi 10000, Vietnam; tuan.levan@hust.edu.vn

[5] Nano Fusion Technology Research Group, Institute for Fiber Engineering (IFES), Interdisciplinary Cluster for Cutting Edge Research (ICCER), Shinshu University, Tokida 3-15-1, Ueda 386-8567, Nagano, Japan; 08tex101@gmail.com

[6] Department of Chemistry and Materials, Faculty of Textile Science and Technology, Shinshu University, Tokida 3-15-1, Ueda, Nagano 386-8567, Japan; muzamilkhatri@gmail.com

* Correspondence: nam.phanduy@hust.edu.vn (D.-N.P.), qamarkhan154@gmail.com (M.Q.K.); kim@shinshu-u.ac.jp (I.-S.K.)

Keywords: cellulose nanofiber; silver nanoparticle; electrospinning; orange essential oil; antibacterial activity

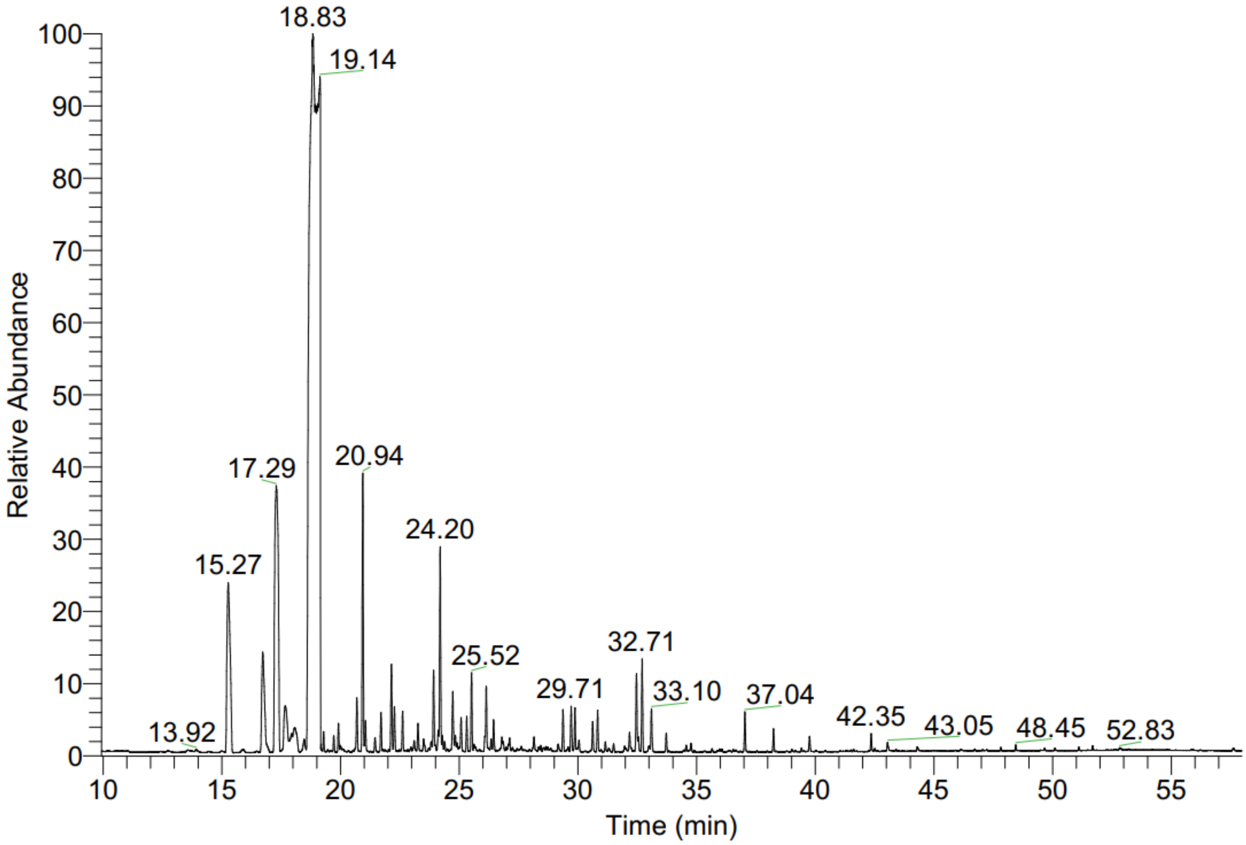

Figure S1. The total ion chromatogram of OEO

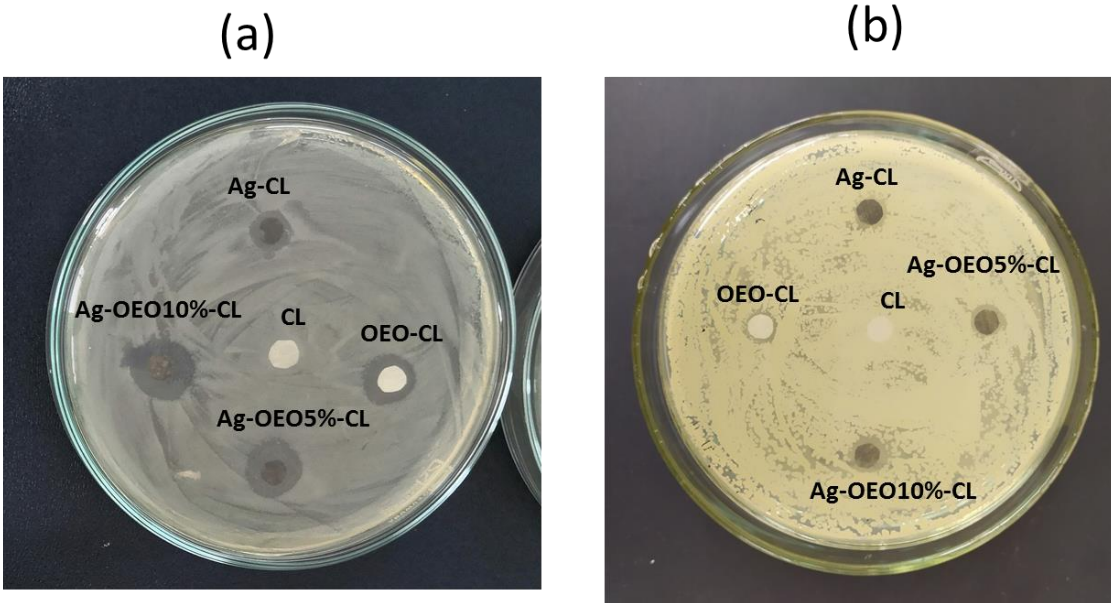

Figure S2. Representative photographs of petri dish of CL - as the negative control, OEO-CL, Ag-CL, Ag-OEO5%-CL, and Ag-OEO10%-CL against (**a**) E. coli, (**b**) B. subtilis
